# Is Your Moss Alive during Active Biomonitoring Study?

**DOI:** 10.3390/plants10112389

**Published:** 2021-11-05

**Authors:** Paweł Świsłowski, Arkadiusz Nowak, Małgorzata Rajfur

**Affiliations:** 1 Institute of Biology, University of Opole, Oleska St. 22, 45-052 Opole, Poland; anowak@uni.opole.pl; 2 Polish Academy of Sciences, Botanical Garden, Centre for Biodiversity Conservation, Prawdziwka St. 2, 02-973 Warsaw, Poland; 3 Institute of Environmental Engineering and Biotechnology, University of Opole, B. Kominka St. 6a, 45-032 Opole, Poland; rajfur@uni.opole.pl or

**Keywords:** active biomonitoring, mosses, chlorophyll content, chlorophyll fluorescence, bioindicator

## Abstract

Biomonitoring was proposed to assess the condition of living organisms or entire ecosystems with the use of bioindicators—species sensitive to specific pollutants. It is important that the bioindicator species remains alive for as long as possible while retaining the ability to react to the negative effects of pollution (elimination/neutralization of hazardous contaminants). The purpose of the study was to assess the survival of *Pleurozium schreberi* moss during exposure (moss-bag technique) based on the measurement of the concentration of elements (Ni, Cu, Zn, Cd, and Pb), chlorophyll content, and its fluorescence. The study was carried out using a CCM-300 portable chlorophyll content meter, portable fluorometer, UV-Vis spectrophotometer, and a flame atomic absorption spectrometer. As a result of the laboratory tests, no significant differences were found in the chlorophyll content in the gametophytes of mosses tested immediately after collection from the forest, compared to those drying at room temperature in the laboratory (*p* = 0.175 for Student’s *t*-test results). Mosses exposed using the moss-bag technique of active biomonitoring were characterized by a drop in the chlorophyll content over 12 weeks (more than 50% and 60% for chlorophyll-*a* and chlorophyll-*b*, respectively). Chlorophyll content in mosses during exposure was correlated with actual photochemical efficiency (yield) of photosystem II (calculated value of Pearson’s linear correlation coefficient was 0.94—there was a significant correlation between chlorophyll *a* and yield *p* = 0.02). The highest metal increases in mosses (*RAF* values) were observed for zinc, lead, and copper after the second and third month of exposure. The article demonstrates that the moss exposed in an urbanized area for a period of three months maintains the properties of good bioindicator of environmental quality.

## 1. Introduction

Bioindicators are indicator species that are used to assess environmental quality and changes occurring over time [1,2]. An example of a bioindicator used on a large scale in biomonitoring studies is moss that meets specific requirements as an indicator plant [3]. Mosses as bioindicators are often used in biomonitoring to assess air pollution by heavy metals [4]. It is necessary to bear in mind that biomonitoring uses living organisms, or parts thereof (tissues), in order to determine the conditions of the environment or the changes that have occurred in it under anthropopressure [5,6]. In biomonitoring with the use of moss to assess air pollution, two methods are distinguished [7]: one is passive biomonitoring that consists of the use of living organisms growing naturally on a given (test) site [8]; the other is a method of active biomonitoring, where living organisms are transferred from their natural habitats and exposed to pollutants on the test site [9]. Air quality biomonitoring that uses mosses is quite a common and frequently used method due to the properties of mosses, including the absence of a root system, uptake of nutrients via the entire body surface from dry and wet depositions, and the fact that the epidermis is most often reduced, while ions deposited on moss surface have direct contact with the exchange points on walls of their cells [10]. An additional advantage of biomonitoring is the low cost and uncomplicated method of obtaining samples, as well as the possibility of complementing/competing with equipment-based monitoring by providing data, using the reaction of indicator species to existing or laboratory environmental conditions [11]. A disadvantage of biomonitoring is the absence of standardization of procedures and techniques used during tests [7,12]. A very important element of active biomonitoring is the sample preparation and all of the procedures occurring before the exposure [13,14]. Depending on the nature of a study, the exposure time of mosses ranges [15] from a few days [16] to even 12 months [17]. However, the question arises: can we say that, before the study and after such a long period of exposure, this material is still a living organism [18]? The measurement of the chlorophyll content is important in physiological and ecological studies of plants, as changes in its content are associated with various key life functions, including growth, photosynthetic capacity, production of metabolites, and responses to environmental stress in higher plants [19]. In the case of mosses, it has been proven that the morphological characteristics of the anatomical structure of mosses’ leaves and the chlorophyll content are related to the photosynthetic activity [20]. The example of treating mosses with wood distillate indicates that the values for the chlorophyll content are related to the maximum quantum yield of primary photochemistry. Results presented in this work do not indicate different values between Fv/Fm and the chlorophyll content [21]. In *Sphagnum* mosses, a positive correlation between the chlorophyll content and the net photosynthesis has been recorded [22]. Environmental factors may affect directly the chlorophyll content in peat moss (temperature, light intensity) [23], and may influence the chlorophyll content in *Sphagna* [24]. Many works have determined the influence of heavy metal accumulation on the chlorophyll content in mosses [25,26]. However, there are no studies demonstrating that, before exposure and when exposed in active biomonitoring over a long period of time and to various types of stresses (e.g., heavy metals), mosses are still alive and thus can still be referred to as bioindicators. Theoretically, in a dry state, mosses are able to survive for a long period of time, even 14 years [27]. However, such an extended period of drought survival had not been confirmed later in the literature. Exposure to, for instance, PAH contamination for a certain period of time causes mosses to spend most of the exposure time in cryptobiosis [28], but there is little research regarding this issue. There is no evidence that, during exposure, moss is still a living organism and, as such, a bioindicator that meets the criteria for organisms intended for biomonitoring. There are also no works that focus on the fact that, before the exposure, moss is also a living organism.

The purpose of our study was to determine to what extent the environmental conditions before the exposure affected moss viability and sorption of heavy metals during active biomonitoring, and how a three-month exposure affects the chlorophyll content and its fluorescence measurements in moss, being a determinant of moss survival under exposure to environmental pollution.

## 2. Results

**Figure 1:**
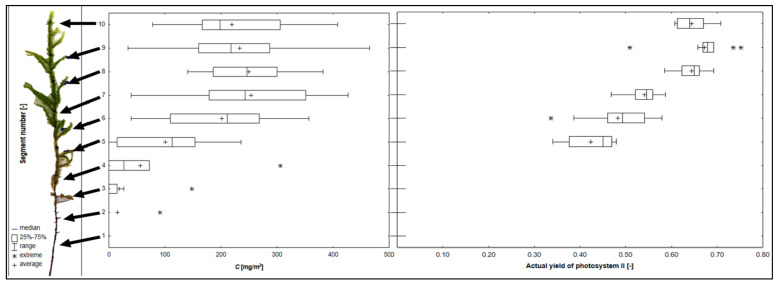
Changes in the chlorophyll content and actual yield of photosystem II in the vertical profile of moss gametophyte.

Figure 1 show the changes in the chlorophyll content and its fluorescence in the vertical profile of *P. schreberi* gametophyte.

Each of the 10 shoots was divided into 10 segments. In the above figure, we can see that there is no chlorophyll in the lower segments, or its concentration is very low (segments 1–4/5). In *P. schreberi*, the average chlorophyll content in segments 7–10 is very similar (219–254 mg/m^2^), with the highest value recorded in segment 9 (465 mg/m^2^). Actual yield of photosystem II was not determined in segments 1–4. In the higher parts, similar to chlorophyll content, the values of actual yield of photosystem II increase, where in segment 9 the highest mean (0.666) and median (0.679) were recorded. For further analyses, mosses were sampled from the higher segments (8–10), which, based on the above results, proved to be the best sites for local measurements of the chlorophyll content and actual yield of photosystem II.

**Figure 2:**
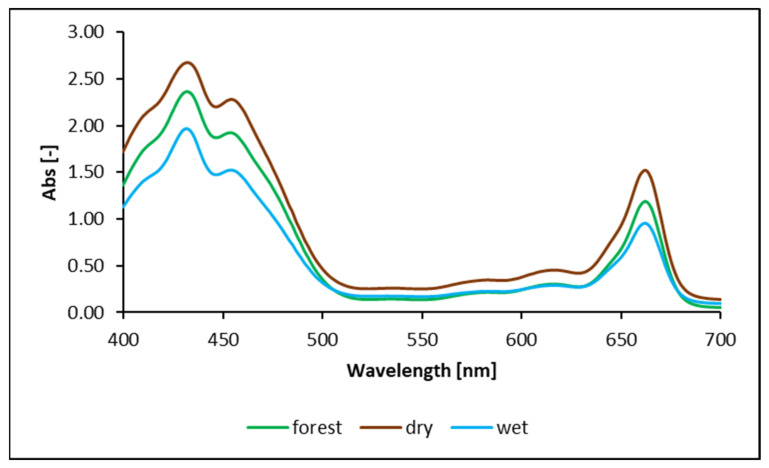
Photoabsorption spectra of pigment from the moss samples.

The experiment conditions/living conditions of mosses have a statistically significant influence on the chlorophyll content in mosses. The upper parts of shoots of mosses were collected for testing based on the results presented in Figure 1. The diagram in Figure 2 shows the spectra obtained from the analysis using the spectrophotometer.

Figure 2 shows the spectra for mosses depending on environmental/laboratory conditions. The spectra are normalized to unity at 662 nm, and arranged at regular intervals of 1 nm. Based on the absorbance values from the diagram in Figure 2, the content of chlorophyll a and b was calculated in gametophytes of the mosses shown in Table 1 below.

The results in Table 1 indicate variable concentrations of chlorophyll *a* and *b* in mosses. The content of this pigment in mosses depends on the living conditions to which they have been exposed. The lowest values according to the spectra shown in the diagram in Figure 2 refer to the mosses dried and sprayed with demineralized water (wet—mosses dried in the laboratory at room temperature and sprayed with deionized water one hour before the analysis). The mean and median values for dried samples (dry—mosses dried in the laboratory at room temperature after collecting them in the forest) are higher than for mosses collected directly from the environment (forest—mosses collected in the forest in situ, examined immediately after collection, fresh samples). Due to the influence of environmental/laboratory conditions on the decreasing chlorophyll *a* and *b* content, mosses can be classified as follows: dried mosses (dry) > mosses collected directly from the forest (forest) > mosses dried and sprayed with demineralized water (wet). For actual yield of photosystem II, the highest values were recorded for forest > dry > wet mosses, the same as for mean values. Nevertheless, the Mann–Whitney U test showed no differences between them, so forest = dry ≠ wet.

Study results of Table 2 below were analyzed using the Student’s *t*-test, indicating statistically significant differences between the content of chlorophyll *a* and chlorophyll *b* in mosses, depending on their living conditions.

**Figure 3:**
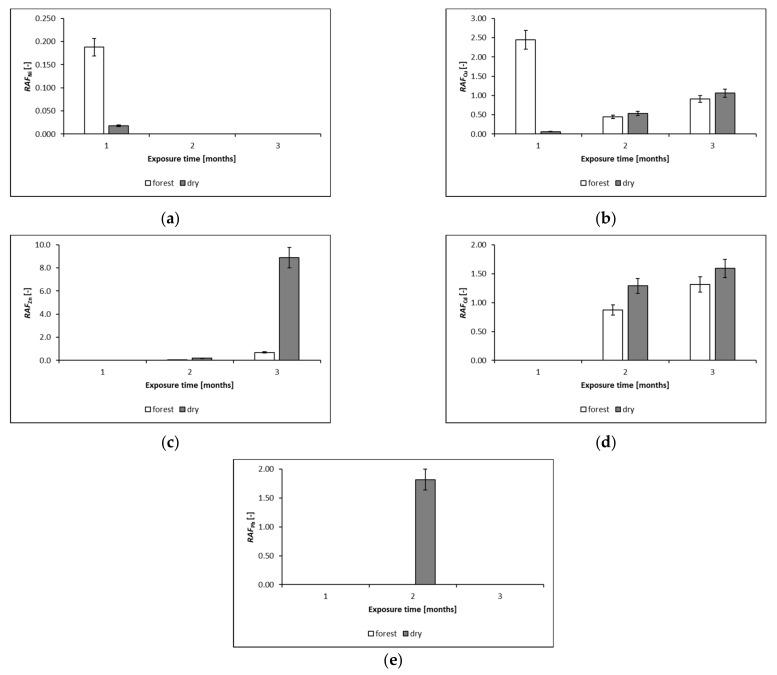
*RAF* values for: (**a**) nickel-, (**b**) copper-, (**c**) zinc-, (**d**) cadmium-, and (**e**) lead-exposed forest (fresh) and dried mosses. *RAF—Relative Accumulation Factors* (meaning and formula are given in Section 4.2).

Results shown in Table 2 indicate that the experiment conditions/living conditions of mosses have a statistically significant influence on the chlorophyll content in mosses. Due to the absence of statistically significant differences between chlorophyll *a* and *b* content in the mosses collected directly from the forest, and in those dried in laboratory conditions, an experiment was carried out in which active mosses collected directly from the forest (forest) and the ones dried in the laboratory at room temperature (dry) were exposed for a period of three months as part of active biomonitoring. A comparison of the ability of fresh and dried mosses to accumulate heavy metals (Ni, Cu, Zn, Cd, and Pb) was carried out. The diagram below in Figure 3 shows *RAF* values for the exposed mosses.

The results presented in Figure 3 show that, for most of the analyzed elements, the relative metal accumulation occurs after 2–3 months, and is higher in mosses dried at room temperature: the conditions under which the mosses were before the exposure affect the level of the analyte accumulation. It has been shown that mosses as bioindicators (dried and still alive (Figure 2) of atmospheric aerosol contamination with heavy metals may accumulate analytes into their cells [29,30].

Demonstrating that dried mosses accumulate heavy metals better, and due to the lack of statistically significant differences in chlorophyll *a* and *b* content between moss samples collected directly from the forest and those dried at room temperature, moss samples dried at room temperature for up to 12 weeks of the experiment were used in order to check the influence of the exposure time and of the exposure to environmental stress caused by the presence of heavy metals in the air on the chlorophyll content and its fluorescence of their gametophytes, to verify moss as a bioindicator of pollutants as a function of time (Table 3).

As we can see in Table 3 above, the exposure of mosses to the environmental stress, such as pollution and changing weather conditions (drying), results in the chlorophyll content decreasing over time. The average chlorophyll *a* content dropped by 54.3%, whereas chlorophyll *b*—by 66.4% after 12 weeks of exposure. The calculated value of Pearson’s linear correlation coefficient r_x,y_ was 0.94—there was a significant correlation between chlorophyll *a* and yield (*p* = 0.02); the coefficient of determination R^2^ was 0.89. For the correlation between chlorophyll *b* and yield r_x,y_ was 0.89—there was a significant correlation (*p* = 0.04) with R^2^ = 0.79.

## 3. Discussion

The structure of the body is not the only factor determining the chlorophyll concentration in the shoot of moss: it also depends on the degree of development and its age [31]. After all, the last, apical (topmost) segments represent a two-/three-year period of moss growth [32]. Other studies also confirm our results for the highest chlorophyll content in the upper parts of the shoot, as the photosynthetically active parts are mainly the upper 4 cm from the top, which constitute the living parts of the moss shoots in *Pleurozium schreberi* [33].

Optical meters are widely used to assess the chlorophyll content in the material in situ, and the measurement value varies within the species, as well as between species, due to the uneven distribution of chlorophyll in the gametophyte [34].

The diagram in Figure 2 shows a typical absorption spectrum of the green moss leaf extract containing a mixture of chlorophyll *a* and *b* [35,36]. Due to the thin leaf elements of the moss, the chlorophyll content in leaves is very low in to vascular plants [37]. We should note that, despite higher values for dry mosses, the results of the Student’s *t*-test in Table 2 indicate that there are no statistically significant differences between mosses in situ and those dried at room temperature. This demonstrates that drying at room temperature does not adversely affect the chlorophyll content of mosses. The study of chlorophyll fluorescence is a valuable tool in ecophysiology, and fluorescence emission spectra are influenced by the chlorophyll content per leaf area [38]. In studies concerning moss sensitivity to prolonged simulated nitrogen deposition, small quantities of added N did not affect the chlorophyll fluorescence. Both the actual photochemical efficiency [Y (II)], as well as the maximum quantum yield (Fv/Fm) parameters were accompanied by specific quantities of chlorophyll. Therefore, the relevant chlorophyll concentration corresponded to the chlorophyll fluorescence (Figure 1 and Table 3), which was an indication of the viability of the moss species [39]. Other studies have proven that the potential rates of photosynthesis, at different depths, in layers of moss turf, were highly correlated with the chlorophyll content [40]. Therefore, a certain chlorophyll content with fluorescence measurements provides information about the vitality of the moss, as it is correlated with other values concerning its viability. The chlorophyll content is related to mosses “being alive”, and is connected with its vital functions. Laboratory tests showed that a decreasing chlorophyll content with increasing Cu and Cr concentrations did not result in the maximum quantum yield PSII (Fv/Fm) changing significantly. The excess of Cu and Cr caused PSII photoinhibition; however, studies on ultrastructure and morphology of the chloroplast demonstrated that no major ultrastructural changes were observed: cells were still alive, even at the highest metal concentrations (100 µM) in solutions [41]. Other studies demonstrated that, with increasing metal concentrations and decreasing chlorophyll (as a reaction to the analyte), chlorophyll fluorescence parameters Fv/Fm and PSII also dropped. Nevertheless, at such high concentrations as e.g., 50 µM Hg, *Eurhynchium eustegium* and *Taxiphyllum taxirameum* mosses still showed a low activity of vital functions. Moss damage caused by the analyte was not correlated with the metal accumulation level [42]. Therefore, it should be considered that the study of the chlorophyll content with fluorescence measurements reflects the viability of mosses and indicates that, during the study, mosses show characteristics of a living organism.

The heavy metal content of moss depends on such factors as contamination of the sampling site, integrity of the plasma membrane, or the type of element and its location in the cell fraction [43]. There are a number of other factors that affect the sorption of metals, but appropriate methods of moss preparation before their exposure seem to be very important [44,45]. Some metals, such as Cu, Zn, or Ni, are essential microelements for plants [46,47]. *P. schreberi* has a high capacity to accumulate especially Cu [48], hence the high *RAF* value in the first month of exposure in the moss samples collected from the forest compared to the control sample. The absence of an epidermis and cuticle makes the mosses capable of absorbing water and pollutants from precipitation that has accumulated on their surface due to dry deposition [49]. For the species under the study, the concave leaf blade causes the ionic forms of the elements dissolved in the precipitation to collect as a bottom pool in the leaf, which promotes bioaccumulation. The absorption of toxins (ectohydrity) in these parts of the leaf is due to the anatomical composition, owing to the absence of the upper protective layer: the drainage layer. Additionally, such features as leaf thickness, the spiral arrangement of the gametophyte leaves on the stem, or the shape of the leaf blade affect the intensity of bioaccumulation due to the easier possibility of and the large area of accumulation of heavy metals [50]. These elements are missing in dried samples, as partial damage resulting from drying [51] causes the analytes to deposit mainly on their surface over time (2–3 months). Furthermore, a lower initial analyte concentration in the control sample before exposure does not always result in a higher *RAF* value [52]. Calculated *RAF* values shown in the diagram in Figure 3 indicate that, for active biomonitoring studies, it is better to use dry moss samples that remain a living material when dried at room temperature. Studies confirm that living transplants generally do not accumulate more than dead material. Mosses have developed mechanisms of resistance to heavy metal contamination, such as cell wall binding of different non-toxic cations naturally occurring in the cells, cell wall thickening [53], chelating of heavy metals, or high reproductive potential [54]. Moss devitalization eliminates the metabolic contribution in the elemental intake. The oven-drying presented in the study did not significantly change the morphology or composition of moss elements [55]. Other studies confirm the thesis that drying mosses (105 °C) leading to their death did not inhibit their ability to accumulate trace elements. Drying caused damage to membranes and cell walls, thus the cell would become more permeable to heavy metals [56]. Another experiment also indicates that the devitalization of mosses does not inhibit their ability to accumulate pollutants [57]. However, according to our knowledge and the definition of biomonitoring [6], the use of dead, devitalized moss is contrary to the idea of biomonitoring, which is based on the use of living organisms. Therefore, relevant preparation techniques should be selected in order to enable the use of mosses that are still alive and able to accumulate pollutants to which they are exposed [58].

Active biomonitoring with the use of mosses focuses mainly on the determination and analysis of elements accumulated by mosses, and the indication of the sources of pollution emissions [59,60,61,62]. However, we should remember that even a short exposure causes damage to gametophyte cells [18,63], while exposure in heavily contaminated areas, or for a long time, may lead to the death of the biomonitor. After six weeks of exposure, a progressive cytoplasmic disorder was observed using a transmission electron microscope, which led to moss death [64]. Other studies have shown that, after six-week exposure, all of the moss samples were almost dead based on the chlorophyll content and fluorescence testing with TEM observations [63]. The results shown in Table 3 confirm that mosses are homochlorophyllous desiccation-tolerant plants [65,66]. The decrease in the chlorophyll content is the result of the progressive moss contamination, and the exposure of mosses to other stress factors that cause their death. Most analytes deposit on the moss surface [54,67]. The deposition of pollutants on their surface also results from secondary enrichment of the atmospheric aerosol, with pollutants carried by the wind from the soil [68]. The fact that mosses maintain, for the period under study (three months during a period of changing weather conditions), their vitality and ability of continuous sorption of pollutants from the air may be associated with them entering the state of cryptobiosis: throughout their whole life cycle, they are able to vegetate in this way for a very long time [69,70]. However, the reference quoted in the introduction [28] is the first work in the literature on moss biomonitoring that concerns the possibility of moss survival in cryptobiosis in the context of changing vitality. Mosses exposed to PAHs and during the dehydration experiment maintained a minimal photochemical activity up to 80 min. There are no other publications in the literature concerning the moment of mosses’ transition into cryptobiosis, and the fact of remaining in it during the exposure to contaminants.

## 4. Materials and Methods

### 4.1. Material

The species used for this study were the moss *Pleurozium schreberi* Mitten (Pl). They were collected/tested in situ between April and July 2020 from forests in Świętokrzyskie Voivodship, Poland. Moss samples were taken and prepared before exposure as part of active biomonitoring, in accordance with the guidelines [14,71]. Mosses were collected at least 5 m away from the canopy of the trees, so as to not be directly exposed to precipitation (only the green parts of moss were taken) [71].

### 4.2. Methods

The presented study was divided into separate experiments. During the first phase, the homogeneity of moss gametophytes in terms of their chlorophyll content was evaluated. The study was carried out using the CCM-300 portable chlorophyll content meter from Opti-Sciences, Inc. (Hudson, NH, USA). The analysis was completed in the vertical profile [in 10 sites—segments [72]—10 shoots of moss] (Figure 1). Each measurement was taken in 10 repetitions in order to determine the location on the gametophyte that had the highest chlorophyll content. Chlorophyll fluorescence of photosystem II (actual photochemical efficiency (yield)) was also measured through this method, using the modulated portable fluorometer (Opti-Sciences, Hudson, NH, USA) under ambient light [73]. Mosses were collected in the field, at noon time. Measurements were made with 10 replicates. Relative humidity ranged from 24 to 36%, and the temperature from 20 to 25 °C during the measurements. The data shown in the table are average values from these measurements (Table 1). These measurements were then used to select the appropriate fragment of the shoot of moss for analysis with a spectrophotometer.

In the first experiment, the influence of external conditions affecting mosses on the chlorophyll content in their gametophytes was analyzed. Mosses in situ (forest), mosses dried in the laboratory at room temperature (dry), and mosses dried in the laboratory at room temperature and sprayed with deionized water one hour before the analysis (wet) with a conductivity of 3.581 µS/cm were used in the study. The chlorophyll content in mosses was measured using a Cary 3500 UV-Vis Compact Peltier spectrophotometer from Agilent Technologies (Santa Clara, CA, USA). For analyses on the spectrophotometer, mosses (0.1 g) were divided into small pieces and ground in a porcelain mortar with 5 mL acetone (pure). The centrifuge (MPW-351RH, MPW Med. Instruments, Warsaw, Poland) was first cooled, and then samples were centrifuged in it (10 min, 10,000 rpm). Each measurement was performed in five repetitions. Chl *a* and chl *b* analytical standards (ChromaDex, Los Angeles, CA, USA, certified dye content > 97%) were purchased in liquid form. Based on the obtained absorbance values, the chlorophyll concentrations were calculated using the extinction coefficients and equation [74] using a spectrophotometer at two wavelengths, 662 and 645 nm, for maximum absorption of chlorophyll-*a* and -*b*, respectively.

Chlorophyll-*a* = 11.75A_662_ − 2.35A_645_ (1)

Chlorophyll-*b* = 18.61A_645_ − 3.96A_662_ (2)

The second experiment was the three-month exposure as part of active biomonitoring of the mosses collected directly from the forest and dried in the laboratory at room temperature (dry). Then, 3 g of mosses were packed into nets, and exposed at a height of about 1.50–2.00 m above ground level. The mosses were exposed on the premises of the Institute of Environmental Engineering and Biotechnology, University of Opole, Opole, Poland, near a public road in use. Each month, samples were collected, and heavy metal concentrations were measured. The study was carried out to compare the ability of fresh and dried mosses to accumulate heavy metals (Ni, Cu, Zn, Cd, and Pb). In order to determine the heavy metals, each moss sample, with a mass of 1.000 ± 0.001 g dry mass (d.m.), was mineralized in a mixture of nitric acid (V) and hydrogen peroxide (HNO_3_ 65%: H_2_O_2_ 37% = 10:6 mL) using a Speedwave Four Berghof, DE microwave oven. The mineralization process was carried out at a temperature of 180 °C. Heavy metals were determined using an atomic absorption flame spectrometer (F-AAS) type iCE 3500 (series 3000), manufactured by Thermo Scientific, USA.

In Table 4, the instrumental detection limits (*IDL*) and instrumental quantification limits (*IQL*) for the spectrometer iCE 3500 are presented. The results were converted into 1 kg of sample. Calibration of the spectrometer was performed with a standard solution from ANALYTIKA Ltd. (CZ). The values of the highest concentrations of the models used for calibration (5 mg/dm^3^ for Ni, Cu, Zn, Pb, and 2 mg/dm^3^ for Cd) were approved as linear limits to signal dependence on concentration. Concentrations of metals were determined in solution after mineralization and dilution, and were filtered into volumetric flasks of 25 cm^3^.

In Table 5, concentrations of heavy metals in certified reference materials BCR-482 *lichen*, produced at the Institute for Reference Materials and Measurements, Belgium, are shown.

The *RAF—Relative Accumulation Factors* was used to determine increases of concentrations of the analytes in the exposed mosses samples, as defined in [76]:

(3) $$RAF=\frac{C_{i,1}-C_{i,0}}{C_{i,0}}$$

where: *C_i_*_,1_ is the concentration of an analyte after exposure period [mg/kg d.m.], and *C_i_*_,0_ is the concentration of an analyte before exposure period [mg/kg d.m].

In the last experiment, the chlorophyll content of dried moss samples (dry) exposed in active biomonitoring for 3 months was analyzed. The mosses were exposed in the car park of the Institute of Environmental Engineering and Biotechnology, University of Opole, Opole, Poland. Measurements using a Cary 3500 UV-Vis Compact Peltier spectrophotometer from Agilent Technologies (USA) were carried out at monthly intervals; measurements of fluorescence chlorophyll were also repeated in this experiment.

Microsoft Excel 2016 and STATISTICA ver. 13.3 software were used to process the data. Shapiro–Wilk’s test was used to assess the normality of the variances. The Student’s *t*-test and Mann–Whitney U test (*p* < 0.05) were also used to assess the statistical significance of the influence of conditions on the chlorophyll content in mosses. Correlation analysis was calculated to determine the statistical significance of the relationship between chlorophyll-a and chlorophyll-b content, and the actual photochemical efficiency (yield) of photosystem II.

## 5. Conclusions

In moss biomonitoring studies, it is often forgotten that, by definition, a bioindicator is a living organism, not a chemical adsorbent [77,78]. Therefore, an important role should be attributed to the fact that during the study, or at least before the exposure, moss should be a living matrix: it should be subjected only to the preparatory methods that will not lead to its death. Therefore, and according to the definition of biomonitoring, it is necessary to exclude devitalization that would make moss only a dead adsorbent of analytes.

Active biomonitoring should be carried out with the bioindicator that remains alive (according to its definition as a living organism), and moss exposure for three months, as shown by the presented results; despite causing a decrease in the chlorophyll content in the gametophytes, this does not lead to death of the bioindicator. This parameter was also related to its vital activity in the form of actual photochemical efficiency (yield) of photosystem II. It remains an organism able to accumulate contaminants due to the preserved viability. In the future, biomonitoring studies should pay attention to the control of moss viability during experiments and observations of the transition into the state of cryptobiosis.

## Figures and Tables

**Table 1:** Chlorophyll *a* and *b* content [mg/L] and actual photochemical efficiency (yield) [-] in mosses depending on the living conditions.

		Forest	Dry	Wet
Chl-*a*	min-max	9.56–17.4	10.4–21.0	8.08–11.3
	average	12.7	16.1	10.1
	median	11.9	16.4	10.5
	SD	3.38	3.57	1.38
Chl-*b*	min-max	4.51–7.39	4.30–13.9	4.42–6.35
	average	5.49	8.16	5.25
	median	5.03	7.55	5.11
	SD	1.33	3.25	0.977
Yield	min-max	0.553–0.706	0.371–0.663	0.281–0.627
	average	0.647	0.532	0.503
	median	0.658	0.524	0.573
	SD	0.044	0.116	0.149

**Table 2:** Student’s *t*-test results for chlorophyll *a* and *b* content in mosses.

		Forest	Dry	Wet
Chl-*a*	forest	-	0.175	0.196
	dry	0.175	-	<0.05
	wet	0.196	<0.05	-
Chl-*b*	forest	-	0.163	0.780
	dry	0.163	-	0.125
	wet	0.780	0.125	-

**Table 3:** Average chlorophyll *a* and *b* content [mg/L] and actual photochemical efficiency (yield) [-] in mosses, depending on the time of their exposure.

	”0”	1w	4w	8w	12w
Chl-*a*	10.8	8.69	8.42	8.92	5.86
Chl-*b*	6.52	4.12	4.26	5.00	4.33
Yield II	0.532	0.332	0.350	0.354	0.240

“0”—mosses before exposure; 1w, 4w, 8w, 12w—chlorophyll content and fluorescence measurement after successive weeks of exposure.

**Table 4:** The instrumental detection limits (*IDL*) and instrumental quantification limits (*IQL*) for the spectrometer iCE 3500 [mg/dm^3^] [14,75].

Metal	*IDL*	*IQL*
Ni	0.0043	0.050
Cu	0.0045	0.033
Zn	0.0033	0.010
Cd	0.0028	0.013
Pb	0.0130	0.070

**Table 5:** Comparison of measured and certified concentrations in BCR-482 *lichen* [14].

Metal	BCR-482 *lichen*		AAS (n = 5)		*Dev.* **
	Concentration	Measurement Uncertainty	Average	±*SD* * of the Concentrations	
	[mg/kg d.m.]				[%]
Ni	2.47	0.07	2.16	0.32	−13.0
Cu	7.03	0.19	6.63	0.17	−5.70
Zn	101	2.20	95.1	2.30	−5.50
Cd	0.56	0.02	0.53	0.03	−5.30
Pb	40.9	1.40	38.2	1.00	−6.60

* *SD* standard deviation. ** relative difference between the measured (c_m_) and certified (c_c_) concentration 100%∙(c_m_ − c_c_)/c_c_.

## Data Availability

The datasets used and/or analyzed during the current study are available from the corresponding author on reasonable request.
